# The lncRNA LOC102549805 (U1) modulates neurotoxicity of HIV-1 Tat protein

**DOI:** 10.1038/s41419-020-03033-4

**Published:** 2020-10-08

**Authors:** Bahareh Torkzaban, Kalimuthusamy Natarajaseenivasan, Taha Mohseni Ahooyi, Masoud Shekarabi, Shohreh Amini, T. Dianne Langford, Kamel Khalili

**Affiliations:** Department of Neuroscience, Center for Neurovirology, Lewis Katz School of Medicine at Temple University, 3500 North Broad Street, Philadelphia, PA 19140 USA

**Keywords:** Molecular biology, Neuroscience

## Abstract

HIV-1 Tat is a potent neurotoxic protein that is released by HIV-1 infected cells in the brain and perturbs neuronal homeostasis, causing a broad range of neurological disorders in people living with HIV-1. Furthermore, the effects of Tat have been addressed in numerous studies to investigate the molecular events associated with neuronal cells survival and death. Here, we discovered that exposure of rat primary neurons to Tat resulted in the up-regulation of an uncharacterized long non-coding RNA (lncRNA), LOC102549805 (lncRNA-U1). Our observations showed that increased expression of lncRNA-U1 in neurons disrupts bioenergetic pathways by dysregulating homeostasis of Ca^2+^, mitigating mitochondrial oxygen reduction, and decreasing ATP production, all of which point mitochondrial impairment in neurons via the Tat-mediated lncRNA-U1 induction. These changes were associated with imbalances in autophagy and apoptosis pathways. Additionally, this study showed the ability of Tat to modulate expression of the neuropeptide B/W receptor 1 (*NPBWR1*) gene via up-regulation of lncRNA-U1. Collectively, our results identified Tat-mediated lncRNA-U1 upregulation resulting in disruption of neuronal homeostasis.

## Introduction

Despite successful treatment with combination antiretroviral therapies (cART), human immunodeficiency virus type 1 (HIV-1) infection remains a persistent challenge for health care systems. In particular, even in people with HIV (PWH) taking combination anti-retroviral therapy (cART), HIV-associated neurocognitive disorder (HAND) occurs in ~30% of PWH and is a growing concern with the HIV^+^ aging populaiton^1–5^. Due to its capacity to increase the production of inflammatory factors, alter gene expression, and impair of a variety of crucial signaling cascades, the effects of HIV-1 transactivator of transcription (Tat) protein on neuronal homeostasis have been subject to increasing examination^6–12^. For instance, some studies have documented the Tat’s capacity to induce acute alterations in Ca^2+^ flux, leading to mitochondrial production of reactive oxygen species (ROS)^1,11,13,14^. Along with impairments to neuronal homeostasis, Tat interferes with the host gene expression by interacting with regulatory proteins and promoter binding factors, such as RNA Polymerase II^15,16^. However, the mechanistic of Tat effects on host cell gene expression and the functional consequences of such effects remain largely uncharacterized.

Among different proposed mechanisms, some studies have demonstrated various long non-coding RNAs (lncRNAs) to mediate an interplay between the host gene regulation machinery and HIV expression regulatory network^17–20^. Unlike short non-coding RNAs, lncRNAs are a class of RNAs with epigenetic function to regulate gene expression at the transcriptional rather than the post-transcriptional level^21^. Moreover, it has been demonstrated that expression profiles of lncRNAs encoded by host cells can be altered upon viral infection^22^. Specifically, some recent studies signify the role of lncRNAs in the regulatory network that control HIV genome expression and replication, as well as pathogenesis and disease progression^21,23–28^. Moreover, several reports have revealed that dysregulation of lncRNAs plays a significant role in the development and progression of a variety of neurodegenerative diseases, including Alzheimer’s disease (AD), Parkinson’s disease (PD), Huntington’s disease (HD), multiple system atrophy (MSA) and amyotrophic lateral sclerosis (ALS)^21,29,30^.

The current research investigated the capacity of Tat to alter host cell gene expression, particularly lncRNAs, as well as the functional consequences of such alterations on neuronal homeostasis. Utilizing a combination of molecular, cellular and genetic approaches, our results demonstrated Tat-mediated upregulation of lncRNA LOC102549805 (lncRNA-U1), along with its splicing variant (lncRNA-U1_66_). Increased lncRNA-U1 expression was associated with the co-expression of a nearby gene *NPBWR1* (Neuropeptide B/W receptor 1), that encodes a transmembrane receptor involved in efficient energy expenditure^31,32^. Subsequent experiments demonstrated a significant association between the upregulation of lncRNA-U1 and the dysregulation of several homeostatic pathways in primary neurons, as evidenced by increased cytosolic Ca^2+^, the generation of ROS by mitochondria, deficient mitochondrial membrane potential, and decreased ATP production. Moreover, immunoassays confirmed the activation of both autophagy and caspase-3 dependent apoptotic pathways, further illustrating the consequences of Tat-mediated alterations in lncRNA-U1.

## Results

### HIV-1 Tat alters expression of lncRNAs in neurons

To assess the effects of HIV-1 Tat on lncRNA expression, we performed RNA-seq analysis on primary neurons derived from embryonic rat (E18) hippocampal tissue as previously described^33^. Among differentially expressed lncRNAs, those with transcript lengths of greater than 1000 base pairs and above two-fold change in expression (up/down-regulated) were selected for validation.

RT-PCR and qRT-PCR analyses were conducted using primers designed to validate the result of RNA-Seq and to confirm the Tat-mediated differential expression of the selected list of lncRNAs in neurons (data not shown). Of the altered lncRNAs, we focused on LOC102549805 (lncRNA-U1) that showed two to four-fold upregulation in response to Tat.

### Tat induces the long non-coding RNA, LOC102549805, and a novel splicing variant

**Fig. 1:**
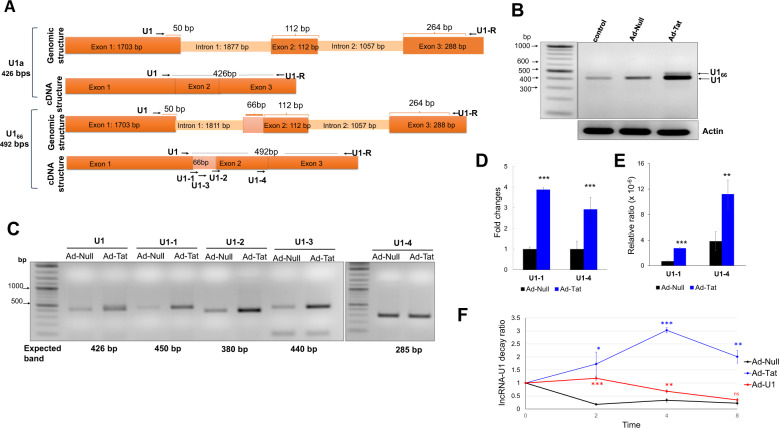
Validation of the Tat-mediated lncRNA-U1 overexpression using RT-PCR and qRT-PCR. **a** Schematic representation of genomic and cDNA structures of lncRNA-U1 isoforms. **b** RT-PCR was carried out using lncRNA-U1 primer pair with the total RNA prepared from Ad-Tat/Ad-Null (MOI: 2) transduced rat primary neuronal culture. Representative agarose gel shows two different bands amplified in HIV-1 Tat samples vs. control. The lower molecular weight (MW) band (U1a) corresponds to lncRNA-U1. The higher (MW) band (U1_66_) corresponds to the minor lncRNA-U1 splice variant. **c** Agarose gel representing RT-PCR results for the exon-specific primers, comparison of the expression of lncRNA-U1 isoforms expression. **d** q-PCR analysis measuring changes in gene expression based on the fold changes in U1_66_ isoform and the whole lncRNA-U1 expression. **e** The relative ratio of basal expression of U1_66_ and total lncRNA-U1 expression. **f** The stability and half-life of lncRNA-U1 was determined using Actinomycin D to inhibit transcription in Ad-Null/Tat/U1 (MOI: 2) treated samples. qPCR analysis following transcriptional inhibition indicated a lncRNA-U1 half-life of 2 h and 4 h in Ad-Null and A-U1 transduced neurons, respectively. However, in Tat expressing neurons, lncRNA-U1 was increased 2–3 folds during this same period (i.e. 2–4 h), and remained elevated throughout the duration of the experiment.

The previously uncharacterized lncRNA LOC102549805 (lncRNA-U1), located on chromosome 5q12 of the rat genome was identified utilizing RGD (RGD: 7711861), as well as NCBI databases^34^. The full-length genomic sequence and established full-length transcript of lncRNA-U1 are 5038 bps and 2104 bps, respectively, and include three exons with 1703, 112, and 288 bps (Fig. 1a top panel, Supplementary Doc.1). Results from RT-PCR using a specific primer set indicated the expression of a minor splice variant for lncRNA-U1 in neurons exposed to either soluble Tat protein (50 ng/ml) or transfected with Adeno-Tat (Fig. 1b and Supplementary Fig. 2). Both amplified fragments using lncRNA-U1 forward (U1F) and reverse primers (U1Rev) were purified from the agarose gel, cloned, and sequenced to verify their specificity. Sequencing confirmed the identity of the amplified fragments and revealed that the novel splice variant contained an additional 66 bps (Fig. 1a bottom panel and Fig. 1b). This variant was referred to as U1_66_, provided that it differs from the identified lncRNA-U1 by 66 bps^34^. Analysis utilizing NCBI’s Basic Local Alignment Search Tool (BLAST) revealed that the extra 66 bps constitute the intronic sequence in the immediate 5′ end of the second exon (Fig. 1a bottom panel, Supplementary Doc.1).

To verify the Tat-mediated alteration of lncRNA-U1, a set of exon-specific forward primers were designed to assess the expression of lncRNA-U1 isoforms in rat neurons exposed to Tat (Fig. 1c). In order to accurately measure both lncRNA-U1 and lncRNA-U1_66_, two different forward primers (U1-1, U1-4) with an identical reverse primer were chosen for expression quantification (Fig. 1c). The U1-1 forward primer quantifies the expression level of the U1_66_ isoform by amplifying the target lncRNA from the exon junction between the first exon and the additional 66 bps (U1_66_) added to 5′ of the second exon and the last exon. The U1-4 primer was designed to quantify lncRNA-U1 expression, regardless of the different splice variants. qRT-PCR indicated that neurons transduced with Ad-Tat demonstrated an increased expression of lncRNA-U1, and/or U1_66_ by ~4-folds (Fig. 1d). Remarkably, the expression of the U1_66_ isoform was nearly undetectable compared to lncRNA-U1 in control samples (i.e. untreated neurons or Ad-Null transduced neurons) (Fig. 1e). These results demonstrated the capacity of Tat to induce elevated expression, as well as splicing variation, for the target lncRNA-U1 (Fig. 1).

To further investigate variation in lncRNA-U1, we examined its stability following transcriptional inhibition and its expression in response to various Tat mutants. Specifically, we first measured lncRNA-U1 expression in Ad-Tat transduced neurons following transcriptional inhibition with actinomycin D (2 µM). Following 1.5 h of treatment, qPCR revealed lncRNA-U1 levels in untreated neurons was reduced by 50%. Conversely, neurons expressing Tat increased lncRNA-U1 expression up to 50% after 2 h, followed by a decline observed at 4 h post-treatment, suggesting that Tat not only overexpressed lncRNa-U1, but also stabilized it (Fig. 1f). To identify the specificity of Tat mutants in up-regulating or stabilizing lncRNA-U1, Hek-293 cells were transfected with different Tat mutant constructs (i.e. Tat-36, Tat-72, and Tat-86) and lncRNAU1 levels were subsequently measured by qPCR. While Tat-36 and Tat-72 did not induce lncRNA-U1 expression, Tat-86 significantly increased lncRNA-U1 comparable to Ad-Tat (Tat 101) (Supplementary Fig. 2b).

**Fig. 2:**
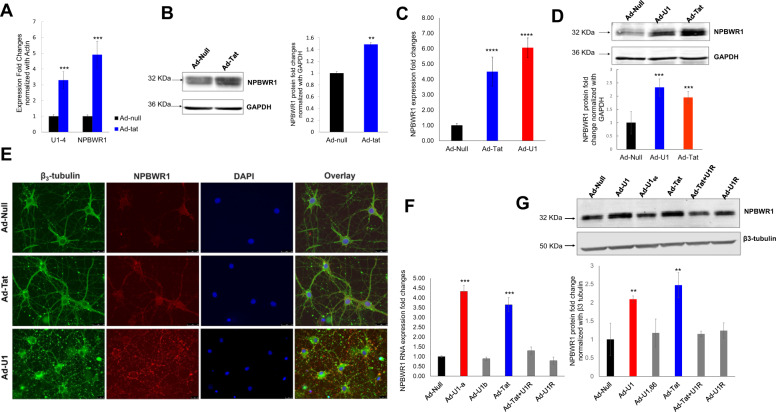
NPBWR1 expression changes. **a** The representative bar chart for the results of the qPCR analysis showed the increased expression of NPBWR1 in rat primary neurons transduced with Ad-Tat (MOI: 2, 72 h). **b** Immunoblotting showed an increase in NPBWR1 protein upon Tat expression. **c** qPCR showed upregulation of NPBWR1 in lncRNA-U1 induced primary neurons, independent of Tat expression. qPCR analysis indicated a 2-fold increased expression of NPBWR1 when primary neurons were transduced with Adeno-U1 (MOI:2) for 72 h. **d** Immunoblotting confirmed the overexpression of NPBWR1 upon lncRNA-U1 up-regulation in primary neurons. **e** ICC confirmed increased NPBWR1 expression in primary neurons upon Tat/U1 treatment, the result of immunoblotting revealed the formation of protein aggregates in the cell borders upon manipulating lncRNA-U1 expression in neurons. **f** q-PCR analysis compared the NPBWR1 expression in neurons transduced with Ad-U1, Ad-U1_66_, Ad-U1R as well as Tat and co-transduction of Tat and U1R revealing a correlation between lncRNA-U1 overexpression and changes in NPBWR1 expression. U1_66_ splice variant did not, however, alter NPBWR1 expression. **g** immunoblotting analysis showed no lncRNA-U1 minor splice variant and confirmed the results from q-PCR analysis.

### lncRNA-U1 is a *cis*-regulator of NPBWR1

Numerous studies have reported that *cis*-regulatory effects of lncRNAs impact transcription of nearby genes^29,35–38^. In this study, we reviewed all recorded genes for quantitative trait loci (QTLs) in the 5q region of the rat genome (RGD:7711861) to assess the potential influence of lncRNA-U1 on nearby genes. QTLs are the genomic regions mapped by identifying a correlation between molecular elements and observed traits^39–41^. Thus, QTL studies usually indicate the association between genetic markers and a target phenotype, which is relevant in pathological studies. Therefore, the list of the genes belonging to QTLs on chromosome 5q (i.e. the location of lncRNA-U1) of the rat genome was compared to the list of differentially expressed genes identified via RNA-seq (Fig. S2). In turn, the neighboring *NPBWR1* (Neuropeptide B/W receptor 1) gene was found to be upregulated by greater than two-fold in response to Tat exposure. *NPBWR1* with a full transcript of 990 bps was located about 211 kbp downstream from the lncRNA-U1 sequence and was the closest coding gene in the lncRNA-U1 vicinity. The result of qRT-PCR analysis confirmed the increased expression of *NPBWR1* when neurons were exposed to either soluble Tat protein or transduced with Ad-Tat for 72 h (Fig. 2a). Western blot analysis validated the significant upregulation of *NPBWR1* upon Tat expression in hippocampal primary neurons (Fig. 2b).

To investigate the potential regulatory effects of lncRNA-U1 on *NPBWR1* gene expression, the full transcripts of lncRNA-U1, the lncRNA-U1_66_, and the U1 reverse complement (U1R) were cloned for delivery into primary rat neurons using adenovirus. Consistent with results in response to Tat, qRT-PCR, and immunoblotting analyses confirmed increased protein expression of NPBWR1 after neurons were transduced with Ad-U1 (Fig. 2c, d). The upregulation of NPBWR1 in response to Ad-Tat or Ad-U1 was also validated via immunocytochemistry (ICC) (Fig. 2e). Notably, these data not only confirmed the increased expression of NPBWR1, but also indicated that lncRNA-U1 overexpression alters the distribution of NPBWR1 from a non-aggregated cytosolic form to condensed aggregates within the neuronal membrane (Fig. 2e). However, U1_66_ had no effect on NPBWR1 RNA or protein levels (Fig. 2f, g). In addition, when lncRNA-U1 expression in neurons was knocked down via co-transduction with both Ad-Tat and Ad-U1R, expression levels of NPBWR1 RNA and protein showed no significant increase (Fig. 2f, g). Consistent with the effect of Tat, these results showed that lncRNA-U1 can directly increase the expression of NPBWR1. Furthermore, knock down of lncRNA-U1 via U1R inhibited Tat’s capacity to increase expression of NPBWR1. Taken together, these results demonstrated the potential regulatory role of lncRNA-U1 on the expression of *NPBWR1* gene.

Although the precise role of NPBWR1 is unknown in vivo, it has been shown to play a role in neuronal homeostasis and metabolism in vitro^31,42^. Specifically, NPBWR1 contributes to cellular responses to hormones and neurotransmitters, and in generating Ca^2+ ^^43^.

### lncRNA-U1 modulates neuronal homeostasis by inducing Ca^2+^ flux

**Fig. 3:**
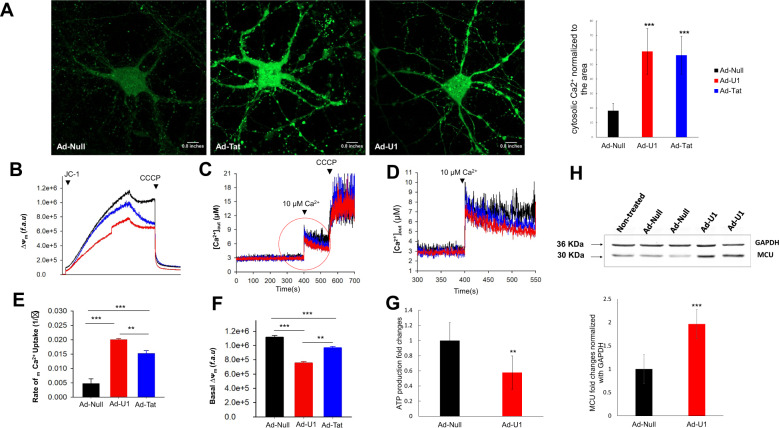
LncRNA-U1 impaired Ca^2+^ signaling in primary neurons. **a** Confocal microscopy using Fluo-4 indicated that Ad-U1 and Ad-Tat transduction increased cytosolic Ca2+ in primary neurons. **b–e** Mitochondrial Ca^2+^ uptake was dysregulated upon the transduction of neurons with Ad-Tat or Ad-U1. Increased Ca^2+^ clearance was observed in neurons expressing Ad-Tat and Ad-U1. **f** Mitochondrial membrane potential Δ*Ψ*m collapsed in neurons transduced with Ad-Tat or Ad-U1. **g** The Ad-U1 neurons had lower levels of ATP production compared to Ad-Null. **h** Immunoblotting showed alterations in the levels of MCU expression in primary neurons upon overexpression of lncRNA-U1.

Given the capacity for lncRNA-U1 to alter NPBWR1 expression, along with NPBWR1’s proposed role in calcium regulation, we next investigated whether lncRNA-U1-mediated increased expression of NPBWR1 led to perturbation in cytosolic Ca^2+^ transients. To measure Ca^2+^ concentration in neurons, the green-fluorescent calcium indicator, Fluo-4, was utilized with live-cell microscopy. A sharp increase in the levels of cytosolic [Ca^2+^]_c_ was observed in rat primary neurons transduced with either Ad-Tat or Ad-U1 (Fig. 3a). To assess whether mitochondria respond to the increase in [Ca^2+^]_c_, we measured mitochondrial Ca^2+^ uptake. Neurons transduced with Ad-Tat or Ad-U1 showed significant increases in mitochondrial calcium uptake [Ca^2+^]_m_ in response to 10 μM Ca^2+^ (Fig. 3b–e). Data also revealed that the mitochondrial membrane potential (Δ*Ψ*_m_) was collapsed (Fig. 3f) and the level of ATP was significantly declined (Fig. 3g) in response to Ad-Tat or Ad-U1.

**Fig. 4:**
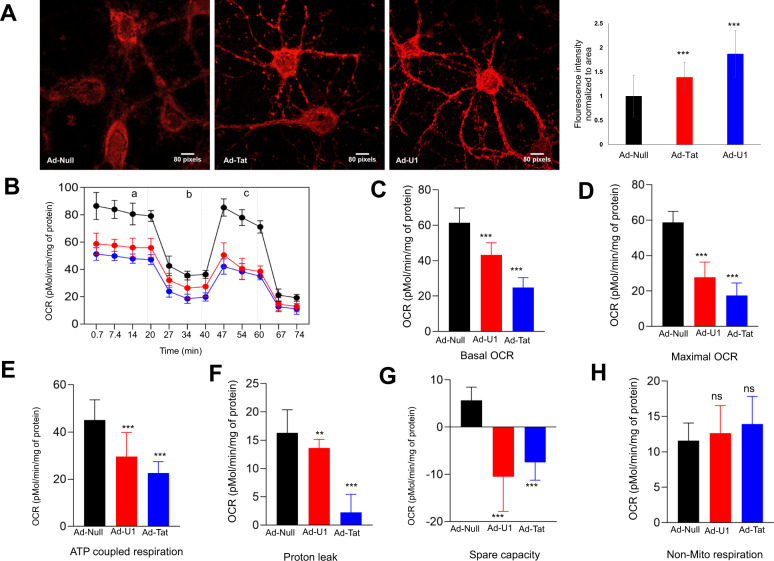
LncRNA-U1 modualtes neuronal respiration and mitochondrial bioenergetics. **a** Confocal microscopy confirmed ROS production upon Ad-Tat and Ad-U1 transduction (red). **b–g** Quantification of basal oxygen consumption rate (OCR), maximal OCR, and ATP coupled respiration, spare capacity of the mitochondria and proton leak show impaired mitochondrial respiration in Ad-Tat, Ad-U1 expressing neurons. Ad-Tat and Ad-U1 dysregulated mitochondrial bioenergetics. **b** Measurements were conducted after oligomycin (**a**), FCCP (**b**), and rotenone/antimycin A (**c**) were added as indicated by dashed vertical lines. **b–g** Measurement of OCR in neurons expressing Ad-Tat and Ad-U1 for 48h showed decreased basal OCR, maximal OCR levels and ATP coupled respiration. **h** Non-mitochondiral respiration did not show significant changes in neurons transduced with either Ad-Tat or Ad-U1.

Although the mitochondrial calcium uniporter (MCU) mediates calcium transport into mitochondria, reports suggest MCU-mediated Ca^2+^ uptake might result in elevated mitochondrial ROS (mROS)^44–46^. To assess the potential for MCU to mediate mitochondrial Ca^2+^ uptake that was observed in response to lncRNA-U1, we investigated changes in mROS production. Accordingly, Ad-Tat/Ad-U1 transduced neurons were incubated with Mitosox red reagent and monitored using confocal microscopy for superoxide production in the mitochondrial matrix. Our analyses indicated that the production of mROS in the mitochondria increased significantly in Ad-Tat and Ad-U1 transduced neurons. Specifically, we observed superoxide accumulation in the neuronal processes in both Ad-Tat and Ad-U1 transduced neurons (Fig. 4a). Moreover, western analysis confirmed the increased expression of MCU in response to Ad-U1 (Fig. 3h).

Having demonstrated increased mROS and a dynamic shift in calcium from the cytoplasm [Ca^2+^]_c_ to the mitochondria [Ca^2+^]_m_, we next evaluated six different mitochondrial parameters: mitochondrial O_2_ reduction rate (basal oxygen consumption rate (OCR)), non-mitochondrial O_2_ reduction rate, proton leak, ATP production, maximal respiration, and spare respiratory capacity using the XF96 respiration analysis. In both Ad-Tat and Ad-U1 transduced neurons, significant reductions in basal OCR were noted (Fig. 4b); in addition, blocking proton channels and inhibiting ATP synthesis by oligomycin treatment revealed significant decreases in ATP coupled respiration (Fig. 4c). Similarly, uncoupling oxidative phosphorylation via FCCP indicated maximal OCR was significantly impaired following expression of Tat or lncRNA-U1, while disruption of the electron transport chain with rotenone confirmed these observed alterations in metabolic activity were due to variations in mitochondrial respiration specifically (Fig. 4d–h). Moreover, analyses of Ad-Null transduced samples confirmed the validity of Tat or U1 induced mitochondrial alterations. Overall, findings suggest that lncRNA-U1 overexpression can induce Ca^2+^ flood in neuronal mitochondria that could be mediated by MCU and are associated with increased mROS. In turn, lncRNA-U1-dependent mitochondrial dysregulation inhibits multiple bioenergetic mechanisms crucial for neuronal homeostasis.

### LncRNA-U1 induces neuronal death via induction of autophagic and apoptotic pathways

**Fig. 5:**
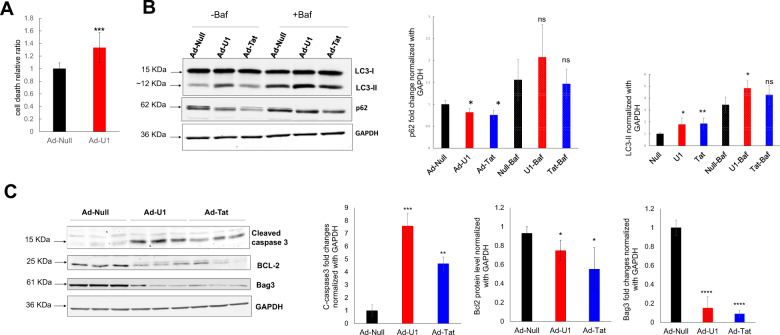
Neuronal survival pathways adversly affected by lncRNA-U1 overexpression. **a** SytoxGreen assay indicated increased cell death in the Ad-U1 transduced neurons. **b** Immunoblotting showed autophagy activation as determined by increased LC3-II and decreased p62, when neurons expressed Ad-Tat or Ad-U1, in the absence or presence of bafilomycin A1 50 nM. **c** Immunoblotting showed increased apoptosis in neurons expressing the Ad-Tat or Ad-U1 for 72h. Apoptosis marker cleaved-caspase3 was significantly increased, while the anti-apoptotic markers Bcl2 and BAG3 decreased significantly.

To determine the downstream consequences of lncRNA-U1, cell viability in response to increased lncRNA-U1 was assessed. Initial results using the SYTOX Green assay showed a significant increase in cell death in neurons transduced with Ad-U1 (Fig. 5a). In addition, western blot analysis confirmed the activation of the autophagy pathway as indicated by increased LC3-II and decreased p62 in neurons transduced with Ad-U1, and to a lesser degree Ad-Tat (Fig. 5b). Moreover, increased levels pro-apoptotic proteins (i.e. cleaved caspase-3) and decreased anti-apoptotic proteins, (i.e. Bcl-2 and BAG3) were observed in neurons transduced with Ad-U1 or Ad-Tat (Fig. 5c). Taken together, these data suggested lncRNA-U1 induced neuronal death by both autophagic and apoptotic pathways.

### In vivo validation of lncRNA-U1 expression in Tat transgenic mice

**Fig. 6:**
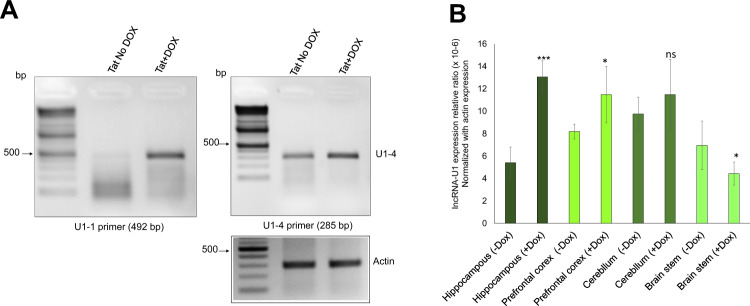
lncRNA-U1 is upregulated in doxycycline-induced Tat transgenic mice. **a** RT-PCR confirmed expression alterations of lncRNA-U1 in the Tat expressing mice. **b** q-RT PCR analysis confirmed increased expression of lncRNA-U1 in three different brain regions (hippocampus, prefrontal cortex, cerebellum) of Tat expressing transgenic mice, the result indicated more significant increases in hippocampus compared to other brain regions. No changes were observed in the brain stem.

To determine if the lncRNA-U1 is a species-specific or a conserved murine lncRNA, we assessed its expression in HIV-1 Tat transgenic mice. RT-PCR analysis of brain tissue from doxycycline (Dox)-inducible GFAP promoter-driven HIV-1 Tat transgenic mice^47^. First, lncRNA-U1 was amplified using the same primer sets for the rat sequence. The RT-PCR amplified fragment was then cloned and sequenced (Fig. 6a) indicating 100% similarity between mouse and rat sequences (Fig. S3). BLAST analyses of the amplified sequence in the NCBI database calculated an 80% similarity between lncRNA-U1 and *Mus musculus* chromosome 1, clone RP23-309D5 sequence^48^. After confirming the expression of lncRNA-U1 in the mouse, total RNA from four different brain regions (hippocampus, prefrontal cortex, cerebellum, and brain stem) of Tat-mice were isolated and utilized for cDNA synthesis. qRT-PCR analyses on the four different Tat transgenic mice treated with DOX versus no DOX confirmed the overexpression of lncRNA-U1 in Tat expressing mice (Fig. 6b). Interestingly, our results revealed that expression levels of lncRNA-U1 were increased in hippocampus and cortex, while we could not determine significant alteration on lncRNA-U1 in the cerebellum. The lncRNA-U1 expression was down-regulated in the brain stem in the presence of Tat. Remarkably, the elevated expression of lncRNA-U1 was greatest in hippocampus followed by the cortex (Fig. 6b).

## Discussion

Given its potential contribution to HIV neuropathogenesis and HAND, it is increasingly relevant to determine the Tat’s capacity to alter neuronal lncRNAs as well as the functional consequences of such alterations on neuronal homeostasis. Findings illustrated a Tat-induced elevation of lncRNA LOC102549805 (lncRNA-U1), along with its splicing variant (lncRNA-U1_66_). Although increased co-expression of a nearby gene *NPBWR1* was also observed in response to Tat, subsequent experiments suggested this pattern of expression might be directly mediated by lncRNA-U1. Furthermore, results suggest that Tat may protect lncRNA-U1 from degradation, provided that increased levels of lncRNA-U1 were observed following transcriptional inhibition. Interestingly, elevated levels of lncRNA-U1 were observed in neurons expressing Tat-86 or full-length Tat (101), but not in response to Tat-36 or Tat-72, suggesting U1 variation is specific to full-length Tat proteins. Furthermore, increased lncRNA-U1 induced the accumulation of mitochondrial Ca^2+^, potentially modulated by MCU, resulting in mitochondrial dysfunction, as indicated by increased mROS, depleted mitochondrial membrane potential and decreased ATP production. Furthermore, the adverse consequences of lncRNA-U1 were associated with decreased neuronal viability, as well as the activation of both autophagy and apoptotic pathways. Thus, our results identified lncRNA-U1 as a novel downstream target for Tat, and demonstrated the consequences of increased lncRNA-U1 in disrupting neuronal homeostasis.

In addition to discovering upregulated expression of lncRNA-U1 in response to HIV-1 Tat, current evidence suggested lncRNA-U1 might also be mediating increased co-expression of the *NPBWR1* gene. Similar to the effect of Tat, exogenous overexpression of lncRNA-U1 directly elevated the expression of *NPBWR1*, while the U1R construct was capable of inhibiting the Tat-induced increase in *NPBWR1*. NPBWR1, a member of G-protein coupled receptors (GPCRs), has been shown to induce Ca^2+^ mobilization from the cytosol to mitochondria^49^. In line with its possible role, our data confirmed an elevated level of [Ca^2+^]_c_ transition to [Ca^2+^]_m_. Results also indicated that this transition could be due in part to MCU, an inward rectifying channel that has a high Ca^2+^- carrying capacity that has been suggested to drive mROS production in response to variations in calcium^44–46,50^. Consistent with this finding, immunoassays demonstrated elevated MCU protein and increased mROS. Thus, data suggest that lncRNA-U1-dependent increased expression of NPBWR1 is associated with variation in the distribution of calcium, with transfer from cytosol to mitochondria potentially modulated by MCU. Our studies highlight the effects of Tat on lncRNA-U1 and indicate novel crosstalk between lncRNAs and GPCRs.

Findings also revealed the functional consequences of lncRNA-U1-mediated dysregulation of calcium. In this context, HIV-1 Tat protein has been shown to alter mitochondrial membrane potential and generate ROS, potentially through alterations in MCU^51^. Moreover, Ca^2+^ accumulation in mitochondria has been shown to impair numerous aspects of neuronal homeostasis, ranging from regulation of ATP production to cell survival pathways^52–54^. Accordingly, our observations revealed that exogenous overexpression of lncRNA-U1 in rat primary neurons modulated basal and maximal oxygen consumption rate, ATP coupled respiration and spare respiratory capacity. In addition, lncRNA-U1 impaired mitochondrial membrane potential and induced significant elevations in mROS.

**Fig. 7:**
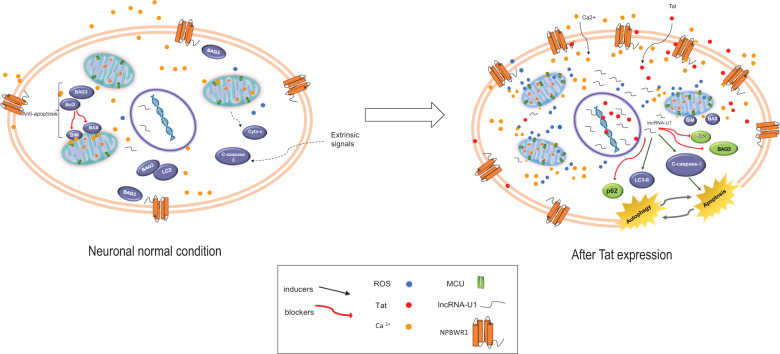
Schematic showing the hypothetical downstream effects of lncRNA-U1 overexpression in primary hippocampal neurons. **a** Schematic representation of normal signaling in neurons before the increased expression of lncRNA-U1. **b** Increased lncRNA-U1 expression alters the neuronal environment by changing the abundance of NPBWR1 followed by increased mitochondrial Ca^2+^ due to increased MCU channel expression. Ultimately, neuronal homeostasis is altered leading to the activation of both apoptosis and autophagy pathways.

Thus, our results suggest a novel mechanism by which Tat stimulates neurotoxicity via elevations in lncRNA-U1 causing an accumulation of mitochondrial calcium, potentially due to activation of NPBWR1 and MCU (Fig. 7). This process is associated with impaired bioenergetic pathways, as well as depleted mitochondrial membrane potential and the elevation in mROS. As a result, imbalances in autophagy and apoptotic pathways ensue, subsequently contributing to compromised neuronal viability.

Consistent with this proposed model, we observed distinct alterations in proteins implicated in autophagy and apoptosis, as well as decreased viability. In particular, lncRNA-U1 significantly decreased BAG3 and BCL-2. This phenomenon is also in consistence with our earlier observation of Tat-induced BAG3 supression^55^. BAG3 is a molecular co-chaperone protein participates in apoptotic inhibition by binding to BCL-2 and forming an anti-apoptotic complex ^56,57^. Conversely, lncRNA-U1 increased cleaved caspase 3, a protein critical for apoptosis^58^. Meanwhile, elevations in LC3-II, crucial modulators of autophagy were observed^59^

Based on the current findings, we conclude that Tat-mediated induction of lncRNA-U1 results in a series of consequences that significantly compromise neuronal homeostasis. Thus, we provide evidence for a novel mechanism of Tat-induced neurotoxicity. Results illustrated that Tat-mediated lncRNA-U1 increase elevate the co-expression of NPBWR1, a GCPR that plays a crucial role in neuronal homeostasis and metabolism by altering Ca^2+^ signaling in neurons. In addition, lncRNA-U1 was found to disrupt neuronal calcium distribution, potentially due to NPWR1 activation, characterized by calcium accumulation in mitochondria, potentially modulated by MCU. Such calcium dysregulation was associated with compromised mitochondrial functioning, impaired bioenergetic pathways as well as the generation of mROS. Consequently, cell viability was significantly impaired. Taken together, these results warrant further examination of the role of lncRNAs, particularly of lncRNA-U1, in mediating the neuropathological processes of HIV. Moreover, understanding these newly discovered pathways could provide novel therapeutic targets for PWH, including those suffering from HAND.

## Materials and methods

### Ethics statement

All protocols followed the guidelines for the use of laboratory animals and were approved by the Institutional Animal Care and Usage Committee at Temple University.

This study used brain tissue of doxycycline induced GFAP promoter-driven HIV-1 Tat transgenic mice generously provided by Dr. Johnny He to the Comprehensive NeuroAIDS Center^47^. Briefly, mice were divided into two groups, (+DOX) and (−DOX) with 4 animals per group. The doxycycline-induced Tat (iTat) animals were injected i.p. with doxycycline hyclate (DOX) (Sigma-Aldrich) at the dosage of 80 mg/kg/day for seven days. The control group was injected with saline (.09% NaCl) in the same manner as their Dox treated counterparts. After seven days, both groups of mice were sacrificed and brains were extracted/dissected into regions of interest (i.e. hippocampus, cerebellum, brain stem, and frontal cortex)^55^. Total RNA was isolated for each brain region using Trizole reagent (Invitrogen, ThermoFisher, Carlsbad, CA), as previously described^55^.

### Rat embryonic cell culture and treatments

Embryonic rat neurons were cultured as described previously^60^. Briefly, the hippocampi were dissected from E18 rat embryos in cold DPBS (Invitrogen). Tissues were then digested using 0.25% trypsin and 1% DNase, at 37 °C for 17 min. The digested tissues were dissociated in warm Neurobasal (Invitrogen) complete medium containing 2% serum, 2% B-27 (Invitrogen), two mM Glutamax (Invitrogen) and 100 U/ml penicillin and 100 U/ml streptomycin. The cells were plated at a density of 2 × 10^6^ per well onto 20 μg/ml poly-d-lysine (PK; Sigma), and 1.2 mg/mL Laminin (Gibco, Thermo Fisher, Carlsbad, CA) coated six-well plates. The media was changed to complete media without serum 24 h after plating. The media was refreshed twice a week for 10 days by replacing 40% of the medium from each well. For treatment, neurons were exposed to HIV-1 Tat protein at 50 ng/ml (101, Immunodiagnostics Inc, Woburn, MA), every 24 h for 48 h. To transduce neurons with viral constructs including Ad-Null, Ad-Tat (101 a.a), Ad-U1, Ad-U1_66_, and Ad-U1R, neurons were incubated for 72 h. To produce viral constructs for transduction, cDNA was cloned from HIV strain 89.6 or amplified lncRNA-U1 into compatible restriction sites of the shuttle plasmid pDC515(IO) and rescued by co-transfecting with pBHGfrtDeltaE1,E3FLP in 293 IQ cells (Microbix Corporation, Mississauga, Ontario Canada). The AD was plaque purified, amplified, and then purified on cesium chloride (CsCl) gradient centrifugation. Plaque purified virus was dialyzed against high salt and MgCl2 for further purification, since CsCl does not generate entirely pure preparations. Collected viral particles diluted for concentration measurement at OD260 and the virus concentration calculated based on the number of particles/ml.

The Ad-U1_66_ and Ad-U1R constructs were prepared and verified by PCR amplification. The Ad-U1_66_ and Ad-U1R full transcripts were cloned into pAdenoG plasmid and packaged as adenoviral vector by Applied Biological Materials, Inc. (Richmond, BC, Canada).

Following treatment, total RNA was extracted using Trizol (ThermoFisher, Carlsbad, CA) extraction protocol followed by RNA cleaning protocol using Direct-zol™ RNA MiniPrep Plus (Zymo Research, Irvine, CA, USA) according to the manufacturer’s protocol. Total RNA sequencing (80 million reads, 2 × 75 PE) was performed by Applied Biological Materials Inc. The RNA-seq analysis was performed as described previously^33^. Briefly, version 6.0 annotation of *Rattus norvegicus* (Rnor 6.0) was applied to identify all non-coding RNAs, including micro-RNAs and lncRNAs. The log_2_ of RPKM (Reads Per Kilobase per Million mapped reads) was used to calculate fold changes for the Tat treated neurons versus untreated control neurons. The full gene and transcript sequences for the selected list of lncRNAs were downloaded from the rat genome database (RGD; http://rgd.mcw.edu)^34^.

To measure the decay rate of lncRNA-U1 in the presence or absence of Tat, primary neurons were transduced with either Ad-Null, Ad-Tat or Ad-U1 for forty-eight hours, and subsequently treated with actinomycin D (Sigma, St. Louis, MO) (2 µM) to inhibit transcription over the course of eight hours. After 8, 4, and 2 h of treatment with actinomycin D, total RNA was extracted and utilized for ensuing qPCR analysis. To determine the rate of decay, lncRNA-U1 expression was normalized to its basal expression in non-treated controls.

The Tat mutant (36,72,86) plasmids were provided by our laboratory resources (Comprehensive NeuroAIDS Center). In brief, each mutant was amplified using plasmid specific primers after cloning to pDC515(IO) plasmid. 293-Hek cells were then transduced with Ad-Tat (Tat-101) and transfected with of each mutant (Lipofectamine 3000; Sigma; NY, USA). Forty-eight h after transfection, cells were lysed and total RNA was extracted for subsequent cDNA synthesis and qPCR analysis.

### Q-PCR validation of selected lncRNAs

cDNA synthesis from total RNA was performed with High Capacity cDNA Reverse Transcription Kit (Thermo Fisher Scientific, Burlington, ONT, CANADA) according to the manufacturer’s protocol. Primers were designed considering the position of exon junctions in the genomic DNA, primer specificity and efficiency were checked by RT-PCR using Q5 High-Fidelity PCR Kit (New England Biolabs, Ipswich, MA). All qPCR reactions were performed with the LightCycler^®^ 96 (Roche, Baltics, UAB) with either the Luna Universal qPCR Master Mix kit (New England Biolabs, Ipswich, MA) or SYBR™ Green master mix (Applied Biosystems, ThermoFisher), according to manufacturer’s protocol. Relative quantification was performed using actin primer reference gene normalization. Primer sequences for amplifying different amplicons of lncRNA-U1 and reference genes are shown in Table S4.

### Gene cloning and sequencing

To confirm the accuracy of amplified fragments using designed primers, RT-PCR products for each primer was separated on 2% agarose gel and the fragments were extracted using the QIAquick Gel Extraction Kit (QIAGEN, Germantown, MD) and were cloned in Invitrogen One Shot Top 10 chemically component cells. Plasmids were extracted via QIAGEN miniprep kit and submitted for Sanger sequencing (GenWise).

### Isolation of protein and western blot analysis

Neurons in culture were washed with PBS twice and lysed in RIPA buffer (50 mM Tris–HCl, pH 7.5, 150 mM NaCl, 0.5% NP40, 1:100 protease inhibitor cocktail (Calbiochem, San Diego, CA), plus mammalian protease inhibitor cocktail (Sigma-Aldrich, MO, USA). The protein concentration was determined by the Bradford method (BioWorld, Columbus, OH). Equal amounts of proteins were separated on SDS-PAGE and transferred to Odyssey nitrocellulose membrane (Li-Cor, Lincoln, NE) by wet transfer (Bio-Rad, Philadelphia, PA). The following primary antibodies were used for western blotting: anti-GPCR GPR7 antibody for NPBWR1 detection (1:1,000, Abcam, Cambridge, MA), anti-GAPDH (1:2,000, Santa Cruz, sc-32233), anti-BCL-2 antibody (1:1000, Santa Cruz, sc-7382), anti-BAG3 antibody (1:1000, Proteintech, 10599-1-AP), anti-p62 antibody (1:1000, Proteintech, 18420-I-AP), anti-LC3 antibody (1:1000, Cell signaling, 3868), anti-ß3 tubulin antibody (1:1000, Santa Cruz,), anti-cleaved-caspase-3 antibody (1:1000, Cell signaling, 9661), anti-MCU (1:1000, abcam, ab121499).

### Immunolabeling and microscopy

Primary neurons in two-well chamber slides were washed with PBS and fixed in 4% paraformaldehyde for 15 min and blocked in 5% BSA in PBST. The neurons were probed with the following antibodies anti-GPCR GPR7 (1:200) (1:1,000, Abcam, Cambridge, MA), and anti- β3-tubulin (1:500) overnight at 4 °C and washed with cold PBST. Alexa Flour®Secondary antibodies donkey anti-mouse IgG 484 and donkey anti-rabbit IgG 568 has been applied for fluorescent labeling (ThermoFisherScientific, Eugene, OR). VECTASHIELD medium (Vector, Laboratories, Burlingame, CA) was used for DAPI labeling and mounting. Leica fluorescent microscope (Leica Microsystems, IL.) was used for imaging.

### Simultaneous measurement of Ca2+ uptake and Δ*Ψ*m in the permeabilized cell system

Primary neurons were washed in Ca^2+^ free PBS, pH 7.4. Cells (7 × 10^6^ cells) were suspended and permeabilized with 40 μg/ml digitonin in 1.5 ml of intracellular medium (ICM) composed of 120 mM KCl, 10 mM NaCl, 1 mM KH_2_PO_4_, 20 mM HEPES-Tris, pH 7.2 and 2 μM thapsigargin to block the SERCA pump. All measurements were performed in the presence of 5 mM succinate. The simultaneous measurement of ΔΨm and extra-mitochondrial Ca^2+^ ([Ca^2+^]out) clearance as an indicator of [Ca^2+^]m uptake was achieved by loading the permeabilized cells with JC-1 (800 nM) and Fura2-FF (0.5 μM), respectively. Mitochondrial uncoupler, carbonyl cyanide m-chlorophenyl hydrazone (CCCP, 2 μM) was added as indicated to collapse the mitochondrial membrane potential. Fluorescence was monitored in a multi-wavelength excitation dual-wavelength emission fluorimeter (HORIBA Scientific, Piscataway, New Jersy) as described previously^61,62^.

#### Measurement of spontaneous cytosolic Ca^2+^ in live neurons

Embryonic rat neurons were grown on 25 mm glass coverslips and loaded with Fluo-4/AM (30 min) in loading buffer 2% BSA (1X salt solution, 2% BSA, 0.18% m/w dextrose, 1 mM Hepes/NaOH,0.05 mM sulfinpyrazone, 0.003% v/v Pluronic acid)^63^. Cytoplasmic Ca^2+^ was recorded (510 Meta; Carl Zeiss, Inc. Heidelberg, Germany) at 488 excitations using a 63× oil objective. Images have been quantified and analyzed using ImageJ^64^.

#### Measurement of mitochondrial oxygen consumption rate

Rat primary neurons were cultured on 96-well (XF96) microplate at the concentration of 3 × 10^4^ cells per well (Seahorse Bioscience, Illerica, MA) and transduced with Ad-Null, Ad-Tat or Ad-U1. Forty-eight hours after transduction, neurons were subjected to OCR measurement at 37 °C in an XF96 extracellular flux analyzer (Seahorse Bioscience). The XF96 extracellular flux assay kit was calibrated using calibration solution (Seahorse Bioscience) in a non-CO_2_, 37 °C incubator overnight, mitochondrial complexes were sequentially inhibited with a: 10 µM oligomycin, b: 10 µM FCCP, c: 1 µM rotenone/antimycin A. Basal and maximal OCR, ATP coupled respiration, spare capacity and proton leak were normalized to total protein and analyzed.

### Mitochondrial superoxide (mROS) measurement

Twelve-day old neurons grown on 25 mm coverslips (Fisherbrand, Pittsburg, PA) were transduced with AdNull, Ad-Tat or Ad-U1. Forty-eight hours after treatment, cells were incubated with mitochondrial oxygen free radical indicator MitoSOX red (Life Technologies, Eugene, OR) for 30 min at 37 °C. Coverslips were mounted for confocal imaging in an open perfusion microincubator (PDMI-2; Harvard Apparatus). Images were obtained at 561 nm excitation by using a 63× oil objective of Confocal microscope (810; Carl Zeiss, Inc.).

### Cell death assay

Rat hippocampal neurons were grown in 96-well plates (3 × 10^4^ cells/well). Seventy-two hours after transduction with AdNull, AdTat or AdU1, cell death was measured using the SYTOX Green cell death assay (Invitrogen). Briefly, cells were incubated at 37 °C for 15 min and fluorescent emission was assessed at excitation/emission of 504/523 nm via spectrophotometer to determine damaged cells versus live cells.

## Supplementary information

Supplemental figure legends

Supplemental Table 1

Supplemental Figure 1

Supplemental Figure 2

Supplemental Figure3

Supplemental Figure 4

Supplemental Figure 5
