# Supplementary material for: The lncRNA LOC102549805 (U1) modulates neurotoxicity of HIV-1 Tat protein
Source: Cell Death Dis. 2020 Oct 8;11(10):835. doi: 10.1038/s41419-020-03033-4 (PMC7546609; doi:10.1038/s41419-020-03033-4)
Supplement: Supplementary file 1 — Supplemental figure legends [file 41419_2020_3033_MOESM1_ESM.docx]

**July 16, 2020**

**The lncRNA LOC102549805 (U1) modulates neurotoxicity of HIV-1 Tat protein**

**SUPPLEMENTARY FIGURE LEGENDS**

Bahareh Torkzaban, Natarajaseenivasan Kalimuthusamy, Taha Mohseni Ahooyi,

Masoud Shekarabi, Shohreh Amini, T. Dianne Langford, Kamel Khalili†

Department of Neuroscience

Center for Neurovirology

Lewis Katz School of Medicine at Temple University

3500 North Broad Street

Philadelphia, PA 19140

†Corresponding author

Phone: 215.707.4500

Email: kamel.khalili@temple.edu

**SUPPLEMENTARY FIGURE LEGENDS**

**Supplementary Table 1**. Primers used in this study.

**Supplementary Figure 1**. Whole genome sequence of lncRNA-U1 based on the NCBI database; green shows exon 1, red: exon 2 and yellow: exon 3. Purple indicates the extra 66 bp in U1_66_ isoform. The areas with no color represent introns.

**Supplementary Figure 2**. A) the result of q-PCR revealed lncRNA-U1 overexpression upon treatment with recombinant Tat protein (50ng) for 48 hrs. B) the result of q-PCRshowed overexpression of NPBWR1 gene in recombinant Tat protein (50ng) treated neuronal culture.

**Supplementary Figure 3.** A) the representative gel agoros shows that the result of RT-PCR confirmed expression of lncRNA-U1 in Hek-293 human cell line. B) Hek -293 cells transfected with Tat mutant (36-72-86) as well Ad-Tat for 48 hrs. The result of q-PCR showed overexpression of lncRNA-U1 upon expression of Tat full length.

**Supplementary Figure 4**. Workflow diagram showing the procedure for characterizing QTLs in lncRNA-lncRNA-U1 region. Briefly, we compared the results of RNA-seq with the list of genes in each QTL in the same region as lncRNA-U1 to generate a list of differentially expressed genes. The data indicated that NPBWR1 was the closest gene to U1 and was therefore selected for further study.

**Supplementary Figure 5**. Comparison of 450 bp of lncRNA-U1 sequence amplified by lncRNA-U1 primer between rat and Tat-expressing mice, showing interspecies similarity.
