# Supplementary material for: The lncRNA LOC102549805 (U1) modulates neurotoxicity of HIV-1 Tat protein
Source: Cell Death Dis. 2020 Oct 8;11(10):835. doi: 10.1038/s41419-020-03033-4 (PMC7546609; doi:10.1038/s41419-020-03033-4)
Supplement: Supplementary file 2 — Supplemental Table 1 [file 41419_2020_3033_MOESM2_ESM.docx]

**Table 1.**

| **Target gene** | **name** | **DNA oligos**  **3՛** |
| --- | --- | --- |
| **lncRNA-U1** | U1-Forward | 5՛-CAGGGGATTTCTGCTGACTAG-3՛ |
| **lncRNA-U1** | U1-Reverse | 5՛-CAGACATGGGTTCTGAGATCC-3՛ |
| **U1-b** | R-U1-1-F | 5՛-ACAGCAGGAGAGTTCAGTGC-3՛ |
| **U1-b** | R-U1-2-F | 5՛-TCACAGCTCATGTTCACG-3՛ |
| **U1-b** | R-U1-3-F | 5՛-AGTTCAGTGCAGAAAGTGGAGC-3՛ |
| **U1-a & b** | R-U1-4-F | 5՛-ATGCTCTGATGAAGCCAG-3՛ |
| **NPBWR1** | Forward | 5՛-CTGCCTTTTGCGGTATTCGC-3՛ |
| **NPBWR1** | Reverse | 5՛-CTAGCTGGATAGCACGCAGT-3՛ |
| **Actin** | Forward | 5՛-CAGGTCCAGACGCAGGATGGC-3՛ |
| **Actin** | Reverse | 5՛-CTACAATGAGCTGCGTGTGGC-3՛ |
