# Supplementary material for: The lncRNA LOC102549805 (U1) modulates neurotoxicity of HIV-1 Tat protein
Source: Cell Death Dis. 2020 Oct 8;11(10):835. doi: 10.1038/s41419-020-03033-4 (PMC7546609; doi:10.1038/s41419-020-03033-4)
Supplement: Supplementary file 3 — Supplemental Figure 1 [file 41419_2020_3033_MOESM3_ESM.docx]

>NC_005104.4:13163282-13168319 Rattus norvegicus strain mixed chromosome 5,

1 TATAAAAATT GCTTGTTATA GCCATTCAGG GTTGCCTTCT AGTCACTTGC CACAGGAGGT TGATTGAGGA TCAATCCCTG ATGCACCAGA AGAAATAAAT

101 CTCTTGCATT TGCATCAAAT TCCATTCTCA TGTCTCACTT GGGGTGGGAG GAGTGTCTCC CAGTAGATAA GACTCACTTT GAATCTTACA TTTGGTGTGT

201 TGGCCAGCAA GCAAGGCCCC CTATGAGATT TTAAGGAATG GTGGTCGGTG AAACAGCTCA AGCTTGATGT CATGTACCAT GGTTGTCTGT ATCTATGTTG

301 TGTGTATCCA TGTTTTCTAT CTTGCTTTTG GTTTGCTCAT GAGCATCTGT AAGGCTTAGA GAGTCTCTGG CCTTGTACAG TCAAATTGCA GCTGGCAGAC

401 ATGCCTTAAA GTTGTGACCA GGTAAGTCTG TGACTGCAGG GAGTTTGGAG GATGCTTCAT GCCCCCACAC CTGGAGGGAG TCAGGAGGAC TCTGCTCTTT

501 TTGGAACATG TTAGACTCAA TAACAGCATA AGTTGGCCAA GTCACCTGAT TACATGGTTT CTGTGCCCAT GGCAGGGGCA AGCTGTCTGT GTATTGATTG

601 TCTTTCTCTT TGTTTTTGGA CTTCAGTATC AGCAGGAGCC AATCGAGTCT TGCTCTGCAA GGGTGAGCTG TTTGAATTTT GATTGTCTTT CTCTGTGTTC

701 TTGGACTTCG ACTTAAGCTA TTTTTGGACA TGAGACAGAC TGAATCTAAC CTATTAAACA TCCTAGTTAA CCACTGGAGG GAGGTTGGGG AAAGAGGAGA

801 AAACTTGTCA GTAGTAGTAA AGAAGGGAAG GTTAGTTACT TTCTGCTCCT CCAAATTGCC CACCTTTGGA GTAGGGTGGC CACCTGTAGA GACCTTTGAC

901 TTTCAGGTGA TCTAAGAAAT AAAGGAAAGA GTCTTTAGAC TAGCATCCCT CAGCCACCCA GACCAAGTGC CCTATATAGT TACCCAGAAG GATTTAGTAA

1001 AGGACCTCAT CCCTTCCCTT GGATAAAAGC TTTTTTTGTC TCCAAGACTG TCCCAGGTCC TGGTCATGAA GGAGAAGAAT CTCATAGAGA ACAACGAGTC

1101 TGTGAAGAAC ATCATCATCT AGGACCCTTC CACAGCAGTT CTGATACTGA TGGATCCTCC ACCCCCTTAC CTCCCTACCA TGGTCCCAGT TCCCACAGCC

1201 CCTCCTCAAG GATCTCCCTT GAGCCCTCAT ACAGCATGCC TTTATCCCCC ACTTCTTCTG GTCCCAGCAA GGCGGAGGGT GGGGAAGGAC CCTCAGAACT

1301 GGGACTGGCC ATGGGAACCT GGAGCAAAAG GGCCACTTCA TCTGATACAA CAGTGGCCCA CCCCCTCTGA TCCTGCAGGC CCATAGATGA GGATTTCAAT

1401 CAGGCCATGC AATATTAGCC TTTCTTTTCA GCTGACTTGT ATAACTAGAA GGCCCAATAC CCCCCTTTTC AGATAACATC AAGGGGCTGA TTAATCTCAG

1501 ACTGTTTTAT TTACTCATCA GCCCACCTGG GATGATAGCC AACAACTCAT GAGAGTGCTC TTCATGACTG AAGATTGAGA GAAGATAGCA GGAAGCCAGA

1601 AAAAATGTCC CCTCAGATAT GGGGGCACCA ACAATTGACC AGGCTGCCAT TGACAGGGGA TTTCTGCTGA CTAGACCTGA CTGGGACTTT AATACAGCAG

1701 GAGGTAAGGA TCACCTGAAG GTCTATCACC AGATTCCATT GGCAGGGCTC AAGGGAGTTG AAGGATGACC CAGCCATTTG ACCAAGGTAC ATGAAGTTAA

1801 GCAGGGCCAG ATGAGCACCC TTAACCTTTC TAGAGTGGCT CTTGGGAGGA ATTCCACCAA TATACACCCT ATGACCCAAG CAGTGAGGAG CACAAAGCTA

1901 CTGTAACTTT GGCTTTCATA GACTAGACTA GTAGAGATAT CAGGAAAAAG CTTCATGGGC TAGAGGGACT ACAGGATAAA TCTTTGAGGA TTTAGTACAG

2001 GTTTCTGAGA TAGTCTACCA TAACAGGGAG ACAGAGAAGG AGCAAAAAAA AAAAAAAGAG AGAGAGAAAA GAAGATAAAA GGGAGATTGA AAAATATGAC

2101 AGGAAAGGAA TTTACAGAAA ATCTTGGGTA CAGTAGTTAG GGAAAGGAGA GAAGAGAGAC TACAACCAAA GAGAAACAGA AAACCCCTTG AAAAGGACCA

2201 GTGTGCATAT TGCAAGGAGA GGGGCCACTG GGCCTGAAAG TTTCCCAACA ACCAGAAGCC AGGGGCAGGA AATTCCAGGC CTTGGGTAAA GCACCTGCAA

2301 AGGTGAAGGT GTTAGCCCTG AGGGAAGAGA GCAAATAGGG AGGACAGGGT TCAGACTCCC TCCCCAAGCC CAGGGTAACT TTGAAAGTGG GAGGGAAGCC

2401 CAACCAATTC TTGATAAATA CTGGGCCCAA TACTCAGTTC TCCTCCAAAC TGATGGGCCA GTCTCCAGCA AAAAAGGTCC AAGAAGCCAC TGAGACTAAA

2501 CAGTATTCAT GGACTACCTG AAGAATTGTA GACCTGGGTG TGGGCTGGGT ATCCCACTCA TTCATGGTCA TCCCTGACTG CCCTTATCCC TTGCTCAAGA

2601 GATATTTGCT TTCCAAAATG GGATCTCAGA TCCACTTCCT TCCCAAGGGC CATCAACTGA TGGGACCTGC TGAAGAACCT GTCCAGGTTC TTACTATTAG

2701 GCTGGAGGAC AAATATAAAT TGTTAGAGAT TAATACCCAA AAGACAGAGG ATATTGCTTG GTGGCTGGGA AAGTTTCTTC TTACTTGGGC TGAAATGGCT

2801 GGGCTAGGTC ATGTGTATCT ATAGCCTGTG TATGTGGAGC TTAAGACCTA GGTTGATCCT GTCTCAGTGT ACCAGCACTT GATGTCGTGG TGGGCACAAG

2901 AAGGAATTAA GCCACACATC TCATGGCTTC TTCACTTTGG GGTGTTGCTT AGTGCTGATA TGCCTGGAAC ATACTGCTCC TGCCAGATTA GAAGCTGGGC

3001 AGTAATGATT ATCTCCCAGT CCAGCACTTA AGAGATGTAA ACCATGATAG AGTGGCTGAC ATCCACTTTA CAGTGCCTAA CCCTTACCGT CTGTTGAGTT

3101 CTTTGTCACC TTCCAGAATT TGGTACACAG TTTTAGACTT GAAAGGTGCT TTCTTCTGCC TGACCTTGGC ATCCCAAAGT CAAGAATACT TCACCTTTGA

3201 ATGGAAAGCC CCAGAGGATG GGATCACCAG TCAGCTGACA TGGACTAGAT TATCCACTGG TTGGGGTCAA TCTGGCCACC TTGCTGCCCA ATCCCAACTT

3301 GGAGCCACCA CGACACAAGT ATCAAATACT GGAAGAAGCC CATGGGTGGA GGAAAGACCT CTCTGACCGA ACCCTTCCAG GAGCTGAGGC TACCTGGTTT

3401 ACAGACAGGA GAAGTTTTCT TCATGAAGGT CAGATAGGGA GCTGCCGTGG TGGATAGCCC AAATGTCATC TAGGCTGGCT GAAATGCTAA CTCCCCACAC

3501 ACCCCCTTTC TCCACAGTTC AGTGCAGAAA GTGGAGCTAA TTTTCCTTGG ACCTTGGGGC CAGCAAGAAT TTTTTTCACA GCTCATGTTC ACGGGGCTAT

3601 ATACTAAGAG AGAAGACTCA TATCAGAGGG AAAAGAAATA AAAAACAAGC AGGAAATCTT GGAACTCTTG GATGCTCTGA TGAAGCCAGC AACTGTGAGT

3701 ATTATTCATT ACCTGGGACA TCAGAAGCAA TAACTAGGCA GATCAAGTGG CTCAAGAAGT GGCTATGCAG GAGCCTGTCC TGGTTATGGA CCTACAAGAG

3801 ACAACAGCTG GGAAATAGTA CTGGACTAAG AAATGGTCTC AATTAGTATA TACTGAAAAA AAGAGGACTC ACATTGCTAG CCACCCTATC CACTACTGCC

3901 TAGAAAAGCA AAGACAATGG TGCACTCAAG GGAAAAACTA TACTCCCCAG AAAACAAGCT GAGGAATTAC TTGACCAAAT GCATAGATGC AATTAGGGGA

4001 TAAGAAGCTC AGCTAAGCAG TCAAAGGATC TAAGGTATAA GTAATGAACC TTAGGTTTTT AACCAGAGAG ACAGTAGAAC AGTGTAAGGT ATGTCAGCAA

4101 GTAAACGCTT ATACAACCAA GAGAAAACAG GGCAAGAGAC CTAAAAGAGA ACGACCTGGG GTATATTGGG AGGTCGATTT CACAGAAGTT AAGTCAGGAA

4201 AATATGGTTA CAAATGCCTC CTAGTGTTTT CTTTAGATAC TTTCTCTAGA TTGGTTGAGG CTTTCCCTAC CAAGCCAGAA ACAGCTACCC TGGTAGCCAA

4301 GAAGATATTA AAAGAAATTT TCCTGAGGTT CGGGGTGCCC AAGTTAATTG GACAATGGTC CTCCTTTTTT TCCAAGGTAA GTCAGGGATT GGCAAAGATA

4401 TTGAGAACTA ATCAGAAGCT CCATTGTGCA TACTGTTCCC AGAGCTCAGG GCAGGTAGAG AATGAACAGA AACCTAAATT AGACCTTAAT TAAATTGACC

4501 TTGGAGACTG GTGCTGACTG AGTGGTGCTC CTTCCCCAGC CCTGTTCCAA GCCTGGAACA TCCCCTACCA TTTTAACTTG ACCCCTTTGA GATCCTGTTT

4601 GGCTCCCTAA CTCCCTTGGT GTTCCCCCTG AACCATCCCC CTGCCCCCAG GTTTCCCAAC TCATTGATAG GAACCTCTTC TTGAGGCTCC AAGCCTTTCA

4701 GTCAATCCAA CATGAGATTT GGCTCAAACT GTCTTCCTTA CATGCTCCAG GAATGCCAAA GACCACTCAC CATTTCCAGA CTGATGACTG GGTCTACATC

4801 CAGAGGCATC AGGCACAATT GTTGGAACCC TGGTGGAAGA GACCCTTCCT GGTACTGTTA ACCACTTCAA CAGTCCTCAA GGTGGATGAT ATTGCGGCCT

4901 TGGTGTATGT GTTTGGTGTG AAACCTGCAG ACACCCCACT TCCCAGGGAC TCCTGCTGCT GCCATGTGCC CATCAAACCT GGACCATGGT TTGGATCTCA

5001 GAACCCATGT CTGGTAGTTA ACAATTCTCT CTCTCTCT

_
