# Supplementary figures and images for: The lncRNA LOC102549805 (U1) modulates neurotoxicity of HIV-1 Tat protein
Source: Cell Death Dis. 2020 Oct 8;11(10):835. doi: 10.1038/s41419-020-03033-4 (PMC7546609; doi:10.1038/s41419-020-03033-4)

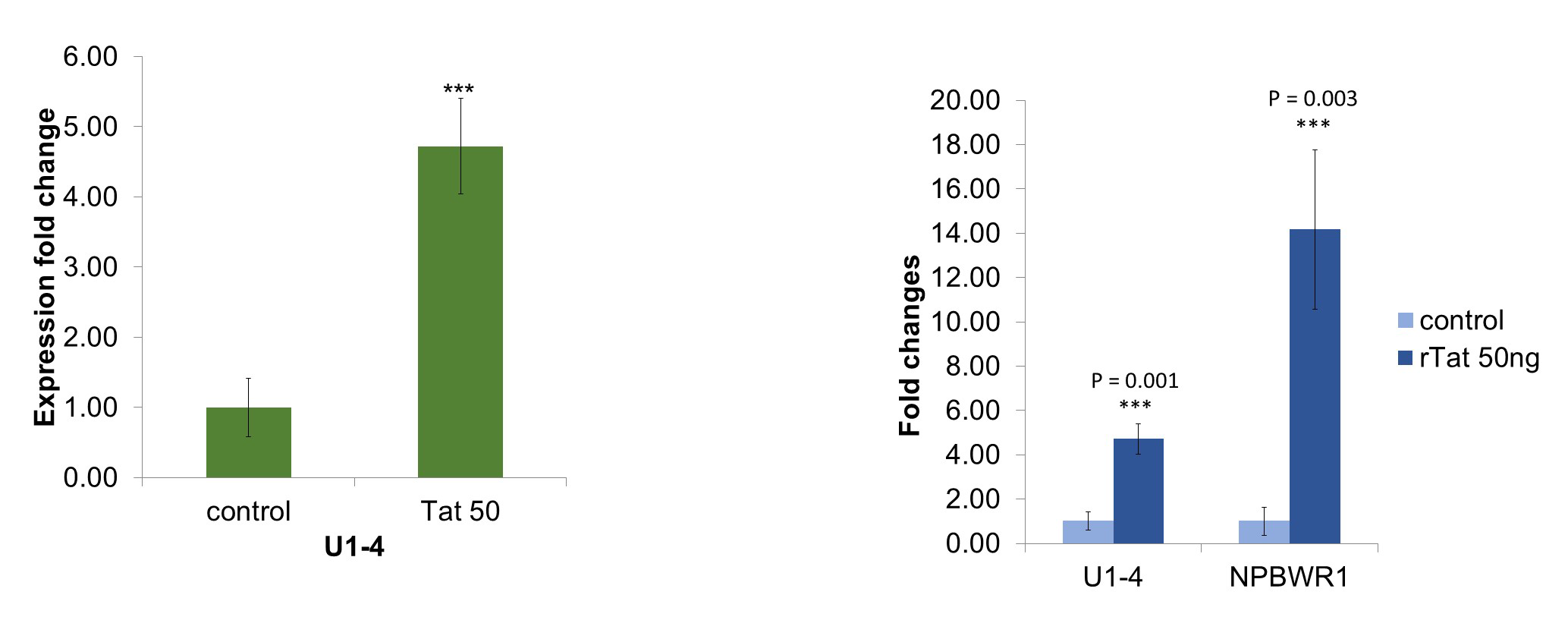

Supplement: Supplementary file 4 — Supplemental Figure 2 [file 41419_2020_3033_MOESM4_ESM.tif]

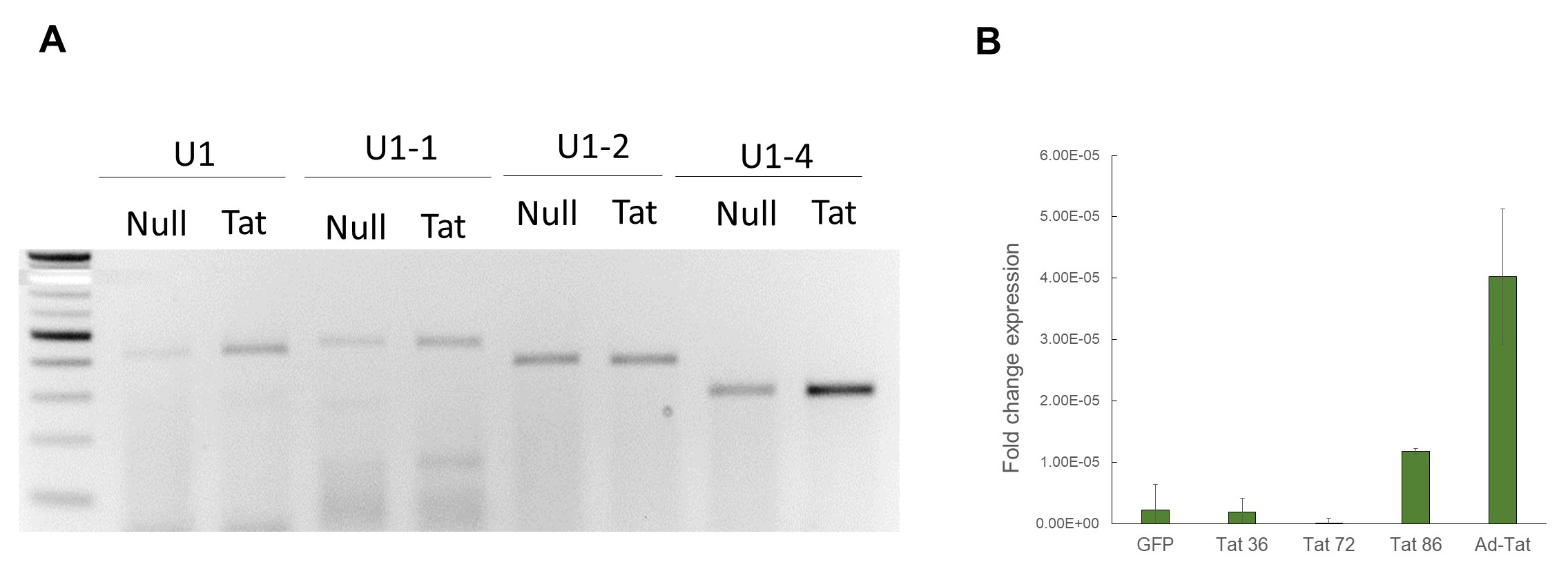

Supplement: Supplementary file 5 — Supplemental Figure3 [file 41419_2020_3033_MOESM5_ESM.tif]

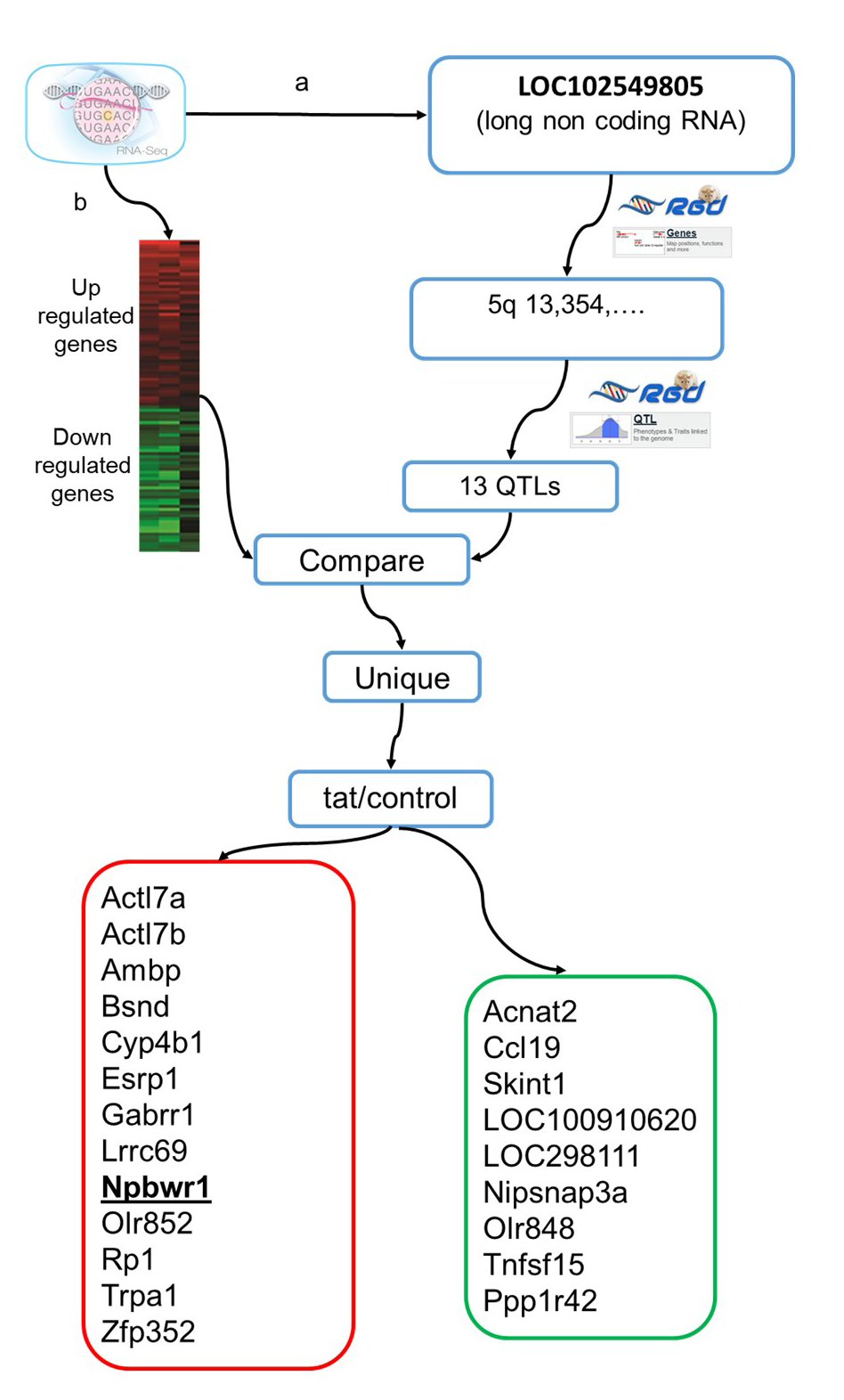

Supplement: Supplementary file 6 — Supplemental Figure 4 [file 41419_2020_3033_MOESM6_ESM.tif]

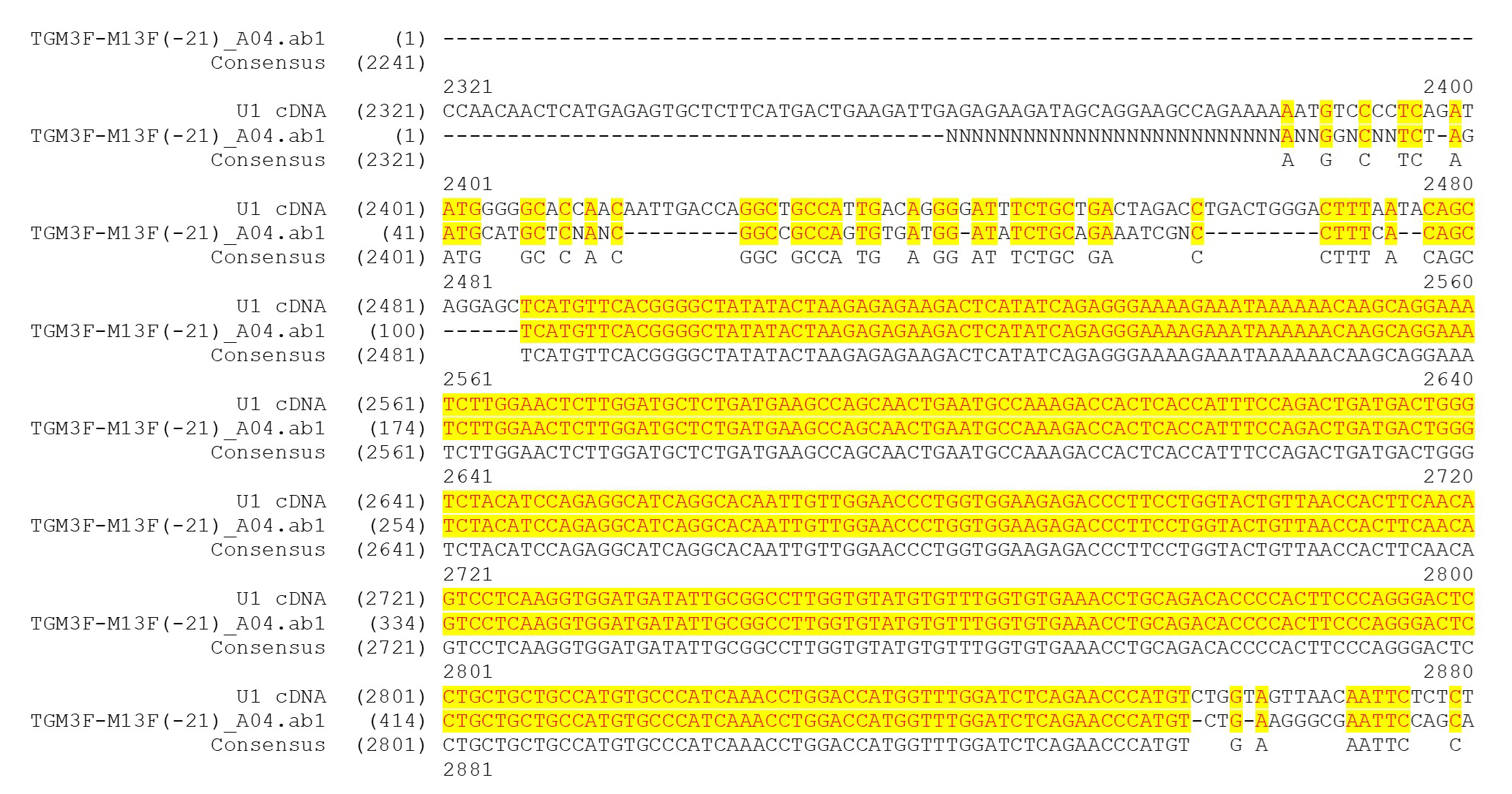

Supplement: Supplementary file 7 — Supplemental Figure 5 [file 41419_2020_3033_MOESM7_ESM.tif]
